# Albumin-to-Alkaline Phosphatase Ratio as a Prognostic Biomarker for Spinal Fusion in Lumbar Degenerative Diseases Patients Undergoing Lumbar Spinal Fusion

**DOI:** 10.3390/jcm11164719

**Published:** 2022-08-12

**Authors:** Youfeng Guo, Haihong Zhao, Haowei Xu, Huida Gu, Yang Cao, Kai Li, Ting Li, Tao Hu, Shanjin Wang, Weidong Zhao, Desheng Wu

**Affiliations:** 1 Department of Spine Surgery, Shanghai East Hospital, School of Medicine, Tongji University, Shanghai 200092, China; 2 Key Laboratory of Inorganic Coating Materials CAS, Shanghai Institute of Ceramics, Chinese Academy of Sciences, Shanghai 200050, China; 3 Institute of Biomedical Engineering, Chinese Academy of Medical Sciences and Peking Union Medical College, Tianjin 300192, China

**Keywords:** degenerative lumbar diseases, albumin-to-alkaline phosphatase ratio, spinal fusion rate, prognostic marker

## Abstract

Objective: To determine if preoperative albumin-alkaline phosphatase ratio (AAPR) is predictive of clinical outcomes in patients with degenerative lumbar diseases undergoing lumbar fusion. Method: 326 patients undergoing posterior lumbar decompression and fusion were retrospectively analyzed. The cumulative grade was calculated by summing the Pfirrmann grades of all lumbar discs. Grouping was based on the 50th percentile of cumulative grade. The relationship between AAPR, intervertebral disc degeneration (IDD) severity, and fusion rate was explored using correlation analyses and logistic regression models. Meanwhile, the ROC curve evaluated the discrimination ability of AAPR in predicting severe degeneration and non-fusion. Results: High AAPR levels were significantly negatively correlated with severe degeneration and non-fusion rate. A multivariate binary logistic analysis revealed that high preoperative AAPR was an independent predictor of severe degeneration and postoperative non-fusion (OR: 0.114; 95% CI: 0.027–0.482; *p* = 0.003; OR: 0.003; 95% CI: 0.0003–0.022; *p* < 0.001). The models showed excellent discrimination and calibration. The areas under the curve (AUC) of severe degeneration and non-fusion identified by AAPR were 0.635 and 0.643. Conclusion: The AAPR can help predict the severity of disc degeneration and the likelihood of non-fusion.

## 1. Introduction

The spine is the central axis bone of the human body and the pillar of the body. It serves as a weight-bearing, shock-absorbing, protective, and moving device and regulates various activities of the upper and lower limbs to maintain body balance. Many patients suffer from severe pain and dysfunction due to degenerative spinal diseases, and their motor ability will be further limited, which is a severe issue in sports medicine. Intervertebral Disc Degeneration (IDD) is a common cause of low back pain and discogenic low back pain, which places a heavy financial burden on families and society [1]. IDD is characterized by extensive morphological and mechanical changes in the disc, decreased intervertebral space height, disc structure destruction, reduced spinal mobility, and loss of disc biomechanical function [2,3]. These changes ultimately lead to low back pain and clinical symptoms. Among the primary biochemical changes associated with IDD are the diminished number and function of nucleus pulposus cells and the loss of matrix macromolecules such as type II collagen and proteoglycan in the extracellular matrix, causing the destruction of cells’ conditions for survival [4,5]. Inflammatory mediators play a crucial role in this process [6]. Research has shown that inflammatory factors promote disc degeneration mainly by triggering an inflammatory response and apoptosis [7]. The inflammatory factors in intervertebral disc tissues interact in a cascade reaction, further aggravating the inflammatory response of intervertebral disc tissue and ultimately accelerating IDD. Spinal fusion is a classic treatment option for those who suffer from degenerative spinal diseases [8]. Some patients, however, suffer from spinal fusion failure after surgery, which leads to a loss of spinal stability and chronic pain caused by local abnormalities, directly affecting the patients’ movement ability. Spinal fusion is a multifaceted and complex process that requires the involvement of many different cells, molecules, extracellular matrix (ECM) components, and growth factors. It is imperative for a successful spinal fusion to undergo the initial phase of the inflammatory process within the first two weeks [9]. Angiogenesis and osteogenesis may be disrupted by repeated excessive inflammation, however, posing a negative impact on bone formation.

The serum albumin (ALB) synthesized in the liver serves various physiological functions, including free radical scavenging, antioxidant, and vascular permeability [10]. Recent reports indicate that ALB is an accurate biomarker of underlying systemic inflammatory responses in organisms [9,10,11]. Additionally, alkaline phosphatase (ALP) is widely expressed in the liver, bones, and kidney and is involved in many physiological processes, such as bone mineralization, vascular calcification, and immune system function [12,13,14]. Studies suggest that biochemical analysis of peripheral blood and whole blood count extraction parameters can aid in the prognosis of diseases such as breast cancer and colorectal cancer [14,15]. The albumin to alkaline phosphatase ratio (AAPR), also based on serum albumin and alkaline phosphatase, can provide insights into systemic inflammation and nutritional status. AAPR has been studied in the context of many different diseases, including non-small cell lung cancer and cholangiocarcinoma, and a low AAPR was associated with a poor prognosis [16,17]. In addition, the activity or mass concentration of bone-specific ALP is closely related to the metabolism of pre-bone cells and forms a chemically classifiable bone matrix. The level of serum ALP activity is a commonly used marker for the evaluation of bone formation rather than the amount of a functional enzyme affecting osteogenesis. Fauran Clavel et al. found that the slowdown of osteogenesis could be demonstrated by the decrease of serum ALP activity [18]. Moreover, serum albumin has been shown to enhance osteogenic differentiation and bone formation in bone defect models [19,20]. Khalooeifard et al. found that increasing protein intake can improve vertebral fusion rate and enhance the recovery ability of patients after spinal fusion [21]. There was also a study showing that ALB decreased the risk of non-fusion rate [22]. As described above, AAPR can be derived or calculated with these two indicators. Moreover, the inflammatory reaction may affect bone metabolism, disrupt the dynamic balance between osteogenesis and osteoclasts, and result in poor bone healing. Hence, AAPR, an indicator reflecting the body systemic inflammatory response, also has the theoretical potential to be an osteogenic marker.

However, there has not been any research on the role of AAPR in degenerative lumbar discs. Therefore, the study of whether AAPR can be applied to lumbar disc degeneration patients is intriguing. Consequently, we conducted a retrospective study to study AAPR’s correlation with the extent of lumbar disc degeneration before surgery and its prognostic value for postoperative fusion rate.

## 2. Materials and Methods

### 2.1. Study Design and Patient Characteristics

Prior to its implementation, ethical approval was obtained from the Shanghai East Hospital Ethics Committee. We enrolled all participants retrospectively and obtained informed consent in accordance with our institutional guidelines.

Three hundred and twenty-six lumbar spinal stenosis patients accompanied with lumbar disc herniation who received lumbar fusion surgery were retrospectively analyzed from May 2019 to May 2020. The following criteria were used to select participants: (1) lower back pain symptoms of lumbar disc herniation and lumbar spinal stenosis; (2) a positive straight leg elevation test or neurological dysfunction (lack of movement, numbness, or lack of reflexes in the lower extremities); (3) MRI findings of disc herniation or spinal stenosis should also correspond to the findings of all participating participants; (4) patients intending to undergo single segmental fusion. Participants who met the following criteria were excluded from the study: (1) a history of spinal deformity, spinal infection, injury, or tumor; (2) the corresponding disc segment had been surgically fused; (3) known history of chronic diseases of lungs, kidneys, or liver; (4) Known inflammatory conditions (such as osteomyelitis, polychondritis, rheumatoid arthritis, etc.).

We collected data on routine clinical variables such as demographics, radiographic findings (intervertebral disc calcification and disc degeneration), and biochemical tests such as uric acid (UA). The presence of intervertebral disc calcification was assessed by lumbar CT. The above information is obtained from our center’s electronic record-keeping system and imaging system. A standard posterior lumbar posterior decompression and fusion was performed in all cases, including instrumentation and bone grafting. Moreover, follow-up radiography was prescribed for the patients after discharge. The imaging system collected lumbar CT data from patients two years after spinal fusion surgery to assess fusion rate. Spinal fusion rate was evaluated by an experienced radiologist without prior knowledge of clinical information through CT images according to the evaluation system proposed by Siepe [23].

### 2.2. Albumin-to-Alkaline Phosphatase Ratio (AAPR) and Disc Degeneration Assessment

We performed routine blood tests on the patient within three days before surgery and recorded relevant data. AAPR is defined as the serum albumin/serum alkaline phosphatase ratio. The Pfirrmann grading system was used by magnetic resonance imaging (MRI) to evaluate the degree of disc degeneration [24], and the cumulative grade is calculated by summarizing the five discs’ grades. All MRI images were read blindly by three experienced spine surgeons. Grouping was based on the median of cumulative grade.

### 2.3. Statistical Analysis

The continuous data were expressed as mean ± standard deviation (SD) and categorical data were presented as frequencies and percentages. The Chi-square and nonparametric tests were used to compare baseline characteristics between groups. Univariate and multivariate analyses were performed by binary logistic regression models to assess the prognostic effect of variables and estimate odds ratios (OR) with 95% confidence intervals (CI). Hosmer–Lemeshow goodness of fit test was used to assess the model fit (Hosmer-Lemeshow statistic ≥ 0.05). The receiver operating characteristic (ROC) curve and the area under the ROC curve (AUC) were also performed to assess the predictive ability of the built models. At the same time, ROC analysis determined AAPR’s predictive power and the optimal cut-off value. *p*-values for linear trends were calculated using the quartile median values. Collected data were encoded into SPSS 26.0 and analyzed.

## 3. Results

### 3.1. Patient Demographics and Outcomes

The baseline characteristics of study participants are shown in Table 1. There were 185 women (56.7%) among the patients with an average age of 63.48. The mean BMI of the patients was 24.80 kg/m^2^. There were significant differences in the following factors: age (*p* < 0.001), ALP (*p* < 0.001), RBP (*p* < 0.001), AST (*p* = 0.048), lumbar CT value (*p* < 0.001), AAPR (*p* < 0.001), fusion rate (*p* = 0.001), and the prevalence of hypertension (*p* = 0.042), disc calcification (*p* < 0.001) and osteoporosis (*p* = 0.018) between the high score group (accumulative grade > 18) and the low score group (accumulative grade ≤ 18). No significant differences were observed among the two groups regarding gender distribution, BMI, VAS, the length of hospital stay, hematological indicators other than ALP, AST and RBP, smoking history, etc.

### 3.2. IDD Severity Classification and Association with AAPR

Table 2 illustrates the distribution of disc grades among the target population. There were 41.7%, 35.9%, and 32.5% of grades for L1/2, L2/3, and L3/4, respectively, which were smaller than 4 (2, 3, and 3). For L4/5 and L5/S1, however, the majority (38.0% and 43.9%) were equal to or greater than 4. At the same time, the low score group showed the same trend as the whole population. On the other hand, all discs except L1/2 scored more than or equal to 4 in the high score group.

We defined mild to moderate degeneration as a score of less than 4 and severe degeneration as a score of more than or equal to 4. As shown in Table 3, the mean levels of LMR were substantially lower in the severe degeneration group (Pfirrmann grade ≥ 4) compared with the mild to moderate degeneration group (Pfirrmann grade < 4) in all lumbar discs except L5/S1. In addition, correlation analysis showed that LMR was significantly correlated with age, osteoporosis, calcification, Scr, VAS, CT value, non-fusion rate, and accumulative grade in all demographic and clinical parameters in Table 4. It is worth mentioning that there is a borderline positive correlation between LMR and UA. The LMR did not show any significant correlation with the length of hospital stay.

### 3.3. Univariable and Multivariable Analysis on Predictive Factors of Severe Disc Degeneration

**Figure 1:**
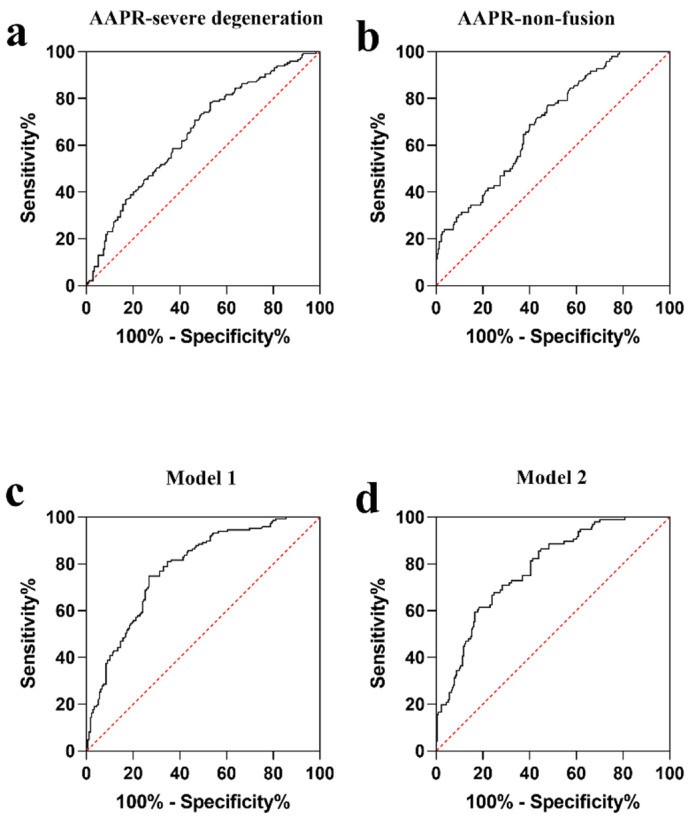
Receiver operating characteristic (ROC) curve to determine the predictive performance of AAPR for severe degeneration (**a**) and non-fusion (**b**). ROC curve analysis of severe degeneration (**c**) and non-fusion models (**d**) (see Table 5 and Table 7 for included variables).

Univariable binary logistic regression analysis based on the entire patient cohort showed that each additional unit of age (*p* < 0.001), hypertension (*p* = 0.043), osteoporosis (*p* = 0.019), calcification (*p* < 0.001), RBP (*p* = 0.001), and AAPR (*p* < 0.001) was significantly associated with severe disc degeneration (Table 5). ROC analysis was performed by defining severe disc degeneration as an endpoint, with the AUC(AAPR) being 0.652 (95% CI: 0.593–0.712) and the difference being statistically significant (*p* < 0.001) (Figure 1a). It is found that the optimal critical value for AAPR is 0.68, while the maximum approximate index is calculated at this point (0.251). Multivariable binary logistic regression model 1 built on clinical parameters further demonstrated that every one unit of increase in RBP (OR: 0.948; 95% CI: 0.919-0.977; *p* = 0.001), AAPR (OR: 0.114; 95% CI: 0.027-0.482; *p* = 0.003), and the occurrence of CHD (OR: 0.360; 95% CI: 0.155-0.834; *p* = 0.017) and disc calcification (OR: 3.215; 95% CI: 1.848-5.594; *p* < 0.001) were determined to be independent predictors of severe disc degeneration. Moreover, AAPR did not interact significantly with calcification, CAD, or RBP in the one-way ANOVA (*p* > 0.05). This model is also capable of calibration and discrimination (*p* > 0.05 and *p* < 0.05, respectively). The area under ROC curve is 0.782 (Figure 1c). At the same time, trend analysis showed that the higher the AAPR, the lower the risk of severe disc degeneration (*p* = 0.010; Table 6).

### 3.4. Univariable and Multivariable Analysis on Risk Factors of Non-Fusion

Univariable binary logistic regression analysis based on the entire patient cohort showed that each additional unit of AAPR (*p* < 0.001) and phosphorus (*p* = 0.002) were significantly associated with postoperative non-fusion rate, as shown in Table 7. ROC analysis was performed by defining non-fusion as an endpoint, with the AUC(AAPR) being 0.695 (95% CI: 0.636–0.755) and the difference being statistically significant (*p* < 0.001) (Figure 1b). The optimal cut-off value for AAPR is 0.63. After adjustment by all covariable estimates, multivariate binary logistic regression analysis model 2 showed that for every one unit increase in UA (OR: 1.005; 95% CI: 1.001-1.010; *p* = 0.014), phosphorus (OR: 16.677; 95% CI: 2.794–99.552; *p* = 0.002), AAPR (OR: 0.003; 95% CI: 0.0003–0.022; *p* < 0.001), and the prevalence of CHD (OR: 0.357; 95% CI: 0.128–0.998; *p* = 0.049) could be independent prognostic factors for non-fusion in patients with lumbar disease undergoing lumbar fusion surgery. At the time, the one-way ANOVA showed no significant interactions between AAPR and UA, CHD, or phosphorus. In addition, this model has effective calibration and discrimination (*p* > 0.05 and *p* < 0.05, respectively). The area under ROC curve of the model is 0.781(Figure 1d). Trend analysis showed that the higher the AAPR, the lower the risk of a non-fusion rate (*p* < 0.001; Table 6).

## 4. Discussion

In this study, we evaluated the prognostic value of AAPR after spinal fusion in patients with lumbar degenerative disease. According to analyses of patient characteristics, AAPR was closely related to non-fusion rate and severe disc degeneration. Binary logistic regression analysis showed that AAPR was an independent predictor of fusion rate and severe disc degeneration in the entire cohorts. Additionally, the levels of AAPR in the severe degeneration group were lower than that in the mild to moderate degeneration group, which verified the close relationship between AAPR and the severity of IDD to a certain extent. In addition, previous studies found that L4/5 or L5/S1 levels are the prone sites for lumbar diseases. In this study, we observed that the degree of disc degeneration was more severe at L4/5 and L5/S1 levels, and the cumulative grade was higher than L1/2, L2/3, and L3/4. At the same time, the ROC curve demonstrated that circulation AAPR levels could be used to predict severe IDD. Therefore, the low AAPR appeared to be an independent risk factor for severe disc degeneration. Additionally, AAPR is not the only factor contributing to disc degeneration and fusion rate. In the logistic regression analysis, serum phosphorus, UA, and CHD were also predictive factors for non-fusion, while the occurrence of CHD, disc calcification, and retinol-binding proteins appeared to have an impact on degeneration.

AAPR incorporates the two basic laboratory parameters, ALB and ALP, which are easily accessible and not too expensive. There is a high concentration of albumin in serum, which serves as a storage and transport system for many endogenous and exogenous substances [25]. It can reflect the human nutritional status and inflammatory state and be related to the severity of many diseases [26,27]. Several studies have demonstrated that ALB regulates inflammatory responses by binding to lipopolysaccharides and reactive oxygen species [28]. Moreover, data have been accumulating on the utility of albumin as a prognostic marker, including the prognostic value of different albumin parameters alone or when combined [29,30]. ALP catalyzes the hydrolysis of phosphate esters and is responsible for transferring phosphate groups, which are mainly produced in the liver and bone. ALP activity could reflect the metabolism and immunity of the body and be used as an immunometric [31,32]. ALP activity is increased in various hepatobiliary diseases, rickets, osteogenesis imperfection, osteomalacia, etc. [33,34]. A higher level of ALP alone has been associated with a poor prognosis. It has become increasingly important to determine the level of alkaline phosphatase in serum in clinical medicine for the detection and monitoring of many diseases. However, there is no study on the correlation between serum ALP and disc degeneration. It is worth mentioning that we separately analyzed the effects of ALB and ALP on severe disc degeneration and fusion rate (see the Supplementary Materials). The results showed that ALP was an independent predictor of non-fusion (OR: 1.047, 95% CI: 1.031–1.063, *p* < 0.001; Supplementary Materials Table S3), not severe disc degeneration (OR: 1.011, 95% CI: 0.999–1.022, *p* = 0.079; Supplementary Materials Table S1). However, Inose et al. found no significant correlation between serum ALP and non-fusion rate [22]. In sharp contrast, ALB is not significantly associated with severe degeneration (OR: 0.976, 95% CI: 0.889–1.072, *p* = 0.612; Supplementary Materials Table S2) and non-fusion rate (OR: 1.020, 95% CI: 0.927–1.122, *p* = 0.685; Supplementary Materials Table S4), which may be related to the sample size. Furthermore, this could be related to the nutritional status of the population included in this study, and there are no primary diseases such as liver and kidney disease, so the groups do not differ significantly. AAPR was applied in patients undergoing surgery for hepatocellular carcinoma for the first time by Chan et al.; this conclusion has been confirmed in the following studies [35,36]. Furthermore, previous research has shown that low levels of this indicator are associated with poor outcomes [37,38]. Despite varying cutoff values, these studies confirm that patients with high AAPR have a better prognosis than those with low AAPR. Therefore, we believe this ratio can provide insight into the microenvironment of local tissue inflammation and can be utilized to measure inflammation status in peripheral blood. It is undeniable that AAPR, a composite index, still has a particular clinical value, even though the effect of AAPR on fusion rate may be due to the mediating effect of ALP.

There were also other factors associated with fusion rate and disc degeneration identified. Multiple binary logistic regression showed that RBP acted as a protective factor against severe disc degeneration, while disc calcification as a risk factor. RBP, a vitamin transporter, is synthesized in the liver and is widely distributed in the blood, cerebrospinal fluid, urinary fluid, and other body fluids. RBP has a complex mechanism of action that exhibits both pro-oxidant and antioxidant effects [39,40]. However, no studies have been conducted on the relationship between RBP and IDD. In addition, intervertebral disc calcification occurs as a result of IDD, and it further aggravates the degeneration [41]. At the same time, the occurrence of calcification was positively correlated with advancing age and a reduced intervertebral height [42]. Calcium deposits in the cellular and extracellular space may cause cell death and decreased activity, resulting in disc degeneration, consistent with this study. A fascinating finding was that although CHD was negatively associated with severe disc degeneration, the effect of CHD on disc degeneration was not significant in models with only CHD and calcification. Furthermore, this study showed that preoperative high serum phosphorus levels were associated with fusion rate, while Shih et al. found no correlation between the fusion rate and the serum levels of calcium or phosphorus [43]. Additionally, we note that UA can enhance fusion rate, which may be related to its antioxidant abilities. Lastly, no significant influence of factors such as age or BMI on disc degeneration or fusion rate was detected, which may be the result of the small sample size.

However, this study has some limitations. Due to the fact that our cohort was a single-center retrospective one containing only Chinese patients, these results may not be generalizable to other populations. Therefore, it is suggested that further multicenter prospective studies be conducted. Additionally, the applicability of the current AAPR cut-off value to other conditions needs to be further examined. At the same time, we investigated the relationship between only one AAPR value and the severity of disc degeneration and fusion rate. Considering that serum AAPR may be affected by other factors, such as liver disease and diet, continuous monitoring may be necessary [44]. Third, larger sample sizes are necessary to test our results, primarily to determine whether or not the statistical significance of results is clinically significant and to measure the smallest clinically meaningful differences. A further research issue is how to exclude the effects of non-steroidal anti-inflammatory drugs, which have been taken by patients before surgery, on AAPR levels in vivo. In addition, since the included population mainly consisted of the elderly with the poor osteogenic ability and the follow-up period was two years, the fusion rate did not meet the expected results. Therefore, we will extend the follow-up period and examine more subtle characteristics of the elderly to verify the validity of this study.

## 5. Conclusions

The results of our study suggest that preoperative AAPR may be a prognostic predictor of postoperative fusion rate. At the same time, AAPR was related to severe disc degeneration, helping clinicians identify high-risk patients and guide individualized treatment.

## Figures and Tables

**Table 1:** Demographic characteristics of patients with disc degeneration disease.

	All	Low Score Group	High Score Group	*p*-Value
		(Accumulative Grade ≤ 18)	(Accumulative Grade > 18)	
Subjects, *n* (%)	326	179	147	
Age (year)	63.48 ± 13.38	60.49 ± 14.93	67.11 ± 10.13	<0.001
Gender				0.562
Male, *n* (%)	141 (43.3)	80 (44.7)	61 (41.5)	
Female, *n* (%)	185 (56.7)	99 (55.3)	86 (58.5)	
BMI (kg/m^2^)	24.80 ± 3.54	24.96 ± 3.49	24.60 ± 3.60	0.371
Smoking (y)	42 (12.9)	22 (12.3)	20 (13.6)	0.724
Alcohol abuse (y)	27 (8.3)	11 (6.1)	16 (10.9)	0.122
Hypertension (y)	155 (47.5)	76 (42.5)	79 (53.7)	0.042
DM (y)	57 (17.5)	30 (16.8)	27 (18.4)	0.704
CHD (y)	40 (12.3)	24 (13.4)	16 (10.9)	0.490
Osteoporosis (y)	115 (35.3)	53 (29.6)	62 (42.2)	0.018
Calcification (y)	164 (50.3)	60 (33.5)	104 (70.7)	<0.001
ALB (g/L)	42.49 ± 3.29	42.56 ± 3.19	42.41 ± 3.41	0.923
ALP (U/L)	74.18 ± 27.27	68.65 ± 24.02	80.90 ± 29.47	<0.001
AAPR	0.64 ± 0.22	0.69 ± 0.24	0.58 ± 0.17	<0.001
Calcium (mmol/L)	2.26 ± 0.10	2.25 ± 0.09	2.27 ± 0.10	0.092
Phosphorus (mmol/L)	1.14 ± 0.17	1.15 ± 0.16	1.14 ± 0.17	0.505
FBG (mmol/L)	5.54 ± 1.47	5.45 ± 1.36	5.65 ± 1.60	0.394
BUN (mmol/L)	6.04 ± 1.77	6.05 ± 1.73	6.02 ± 1.82	0.547
Scr (μmol/L)	72.33 ± 23.22	72.09 ± 21.65	72.62 ± 25.06	0.779
UA (μmol/L)	320.44 ± 82.57	323.42 ± 80.03	316.82 ± 85.69	0.597
ALT (U/L)	19.51 ± 14.34	20.12 ± 16.06	18.77 ± 11.92	0.549
AST (U/L)	19.12 ± 8.64	18.75 ± 9.80	19.56 ± 6.99	0.048
RBP (mg/L)	41.26 ± 9.54	42.83 ± 9.49	39.35 ± 9.27	<0.001
Fusion (y)	230 (70.6)	140 (78.2)	90 (61.2)	0.001
VAS	3.91 ± 1.84	3.73 ± 1.84	4.13 ± 1.84	0.067
Hospital stay (day)	12.21 ± 4.39	12.20 ± 4.36	12.21 ± 4.45	0.854
CT value (HU)	131.26 ± 49.20	140.51 ± 52.51	120.01 ± 42.35	<0.001

Values are expressed as *n* (%) or mean ± SD. BMI, body mass index; VAS, visual analogue scale; AAPR, albumin-to-alkaline phosphatase ratio; FBG, fasting blood glucose; CHD, coronary heart disease; DM, diabetes mellitus; ALT, alanine transaminase; AST, aspartate transaminase; RBP, retinol-binding Protein; UA, uric acid; BUN, blood urea nitrogen; Scr, serum creatinine.

**Table 2:** The Pfirrmann grading system for lumbar disc degeneration.

	1	2	3	4	5
All (*n* = 326)					
L1/2	0	136 (41.7)	106 (32.5)	50 (15.3)	34 (10.4)
L2/3	0	79 (24.2)	117 (35.9)	78 (23.9)	52 (16.0)
L3/4	1 (0.3)	49 (15.0)	106 (32.5)	105 (32.2)	65 (19.9)
L4/5	0	24 (7.4)	82 (25.2)	124 (38.0)	96 (29.4)
L5/S1	0	27 (8.3)	53 (16.3)	103 (31.6)	143 (43.9)
Low score group (*n* = 179)					
L1/2	0	114 (63.7)	47 (26.3)	14 (7.8)	4 (2.2)
L2/3	0	77 (43.0)	81 (45.3)	19 (10.6)	2 (1.1)
L3/4	1 (0.6)	49 (27.4)	90 (50.3)	37 (20.7)	2 (1.1)
L4/5	0	24 (13.4)	64 (35.8)	74 (41.3)	17 (9.5)
L5/S1	0	27 (15.1)	42 (23.5)	65 (36.3)	45 (25.1)
High score group (*n* = 147)					
L1/2	0	22 (15.0)	59 (40.1)	36 (24.5)	30 (20.4)
L2/3	0	2 (1.4)	36 (24.5)	59 (40.1)	50 (34.0)
L3/4	0	0	16 (10.9)	68 (46.3)	63 (42.9)
L4/5	0	0	18 (12.2)	50 (34.0)	79 (53.7)
L5/S1	0	0	11 (7.5)	38 (25.9)	98 (66.7)

Values are expressed as *n* (%).

**Table 3:** The relationship between the severity of individual disc degeneration and AAPR/.

		AAPR	*p*
L1/2	Pfirrmann grade < 4	0.65 ± 0.21	0.003
	Pfirrmann grade ≥ 4	0.59 ± 0.22	
L2/3	Pfirrmann grade < 4	0.68 ± 0.23	<0.001
	Pfirrmann grade ≥ 4	0.58 ± 0.18	
L3/4	Pfirrmann grade < 4	0.70 ± 0.25	<0.001
	Pfirrmann grade ≥ 4	0.58 ± 0.17	
L4/5	Pfirrmann grade < 4	0.68 ± 0.19	<0.001
	Pfirrmann grade ≥ 4	0.62 ± 0.23	
L5/S1	Pfirrmann grade < 4	0.67 ± 0.23	0.181
	Pfirrmann grade ≥ 4	0.63 ± 0.21	

Values are expressed as mean ± SD; AAPR, albumin-to-alkaline phosphatase ratio.

**Table 4:** Correlation of AAPR with demographic and clinical parameters.

	*r*	*p*
Age	−0.110	0.046
Gender	0.070	0.210
BMI	0.028	0.611
Smoking	0.026	0.640
Alcohol abuse	−0.070	0.209
Hypertension	−0.045	0.417
DM	−0.014	0.796
CHD	−0.058	0.295
Osteoporosis	−0.167	0.002
Calcification	−0.422	<0.001
Calcium	0.025	0.656
phosphorus	0.080	0.150
FBG	−0.034	0.546
BUN	0.046	0.408
Scr	0.179	0.001
UA	0.108	0.052
ALT	−0.019	0.728
AST	−0.064	0.249
RBP	0.036	0.521
Non-fusion	−0.132	0.017
VAS	−0.132	0.017
Hospital stay, day	0.087	0.118
CT value	0.198	<0.001
Accumulative grade	−0.379	<0.001

BMI, body mass index; VAS, visual analogue scale; AAPR, albumin-to-alkaline phosphatase ratio; FBG, fasting blood glucose; CHD, coronary heart disease; DM, diabetes mellitus; ALT, alanine transaminase; AST, aspartate transaminase; RBP, retinol-binding protein; UA, uric acid; BUN, blood urea nitrogen; Scr, serum creatinine.

**Table 5:** Univariate and multivariate analysis model 1 of risk factors for severe degeneration.

Variables	Univariate		Multivariate	
	OR (95% CI)	*p*	OR (95% CI)	*p*
Age (year)	1.042 (1.023–1.062)	<0.001	1.027 (0.999–1.055)	0.062
Gender (male)	0.878 (0.565–1.364)	0.562	0.953 (0.478–1.903)	0.892
BMI	0.971 (0.913–1.034)	0.358	0.979 (0.903–1.061)	0.604
Smoking	1.124 (0.587–2.151)	0.724	1.492 (0.623–3.574)	0.369
Alcohol abuse	1.865 (0.837–4.155)	0.127	2.037 (0.735–5.646)	0.172
Hypertension	1.574 (1.015–2.443)	0.043	1.179 (0.649–2.141)	0.588
DM	1.117 (0.630–1.982)	0.704	0.935 (0.397–2.201)	0.877
CHD	0.789 (0.402–1.548)	0.490	0.360 (0.155–0.834)	0.017
Osteoporosis	1.734 (1.096–2.742)	0.019	1.045 (0.581–1.880)	0.883
Calcification	4.797 (2.993–7.689)	<0.001	3.215 (1.848–5.594)	<0.001
AAPR	0.055 (0.015–0.194)	<0.001	0.114 (0.027–0.482)	0.003
Calcium	6.241 (0.649–59.996)	0.113	14.486 (0.796–263.562)	0.071
phosphorus	0.618 (0.166–2.308)	0.474	1.456 (0.296–7.161)	0.644
FBG	1.096 (0.944–1.274)	0.229	1.080 (0.868–1.344)	0.489
BUN	0.988 (0.873–1.118)	0.850	0.975 (0.822–1.157)	0.773
Scr	1.001 (0.992–1.010)	0.839	1.004 (0.988–1.021)	0.590
UA	0.999 (0.996–1.002)	0.472	1.001 (0.997–1.004)	0.755
ALT	0.993 (0.977–1.009)	0.405	0.996 (0.961–1.032)	0.832
AST	1.011 (0.985–1.038)	0.408	1.003 (0.947–1.061)	0.928
RBP	0.960 (0.937–0.984)	0.001	0.948 (0.919–0.977)	0.001

BMI, body mass index; AAPR, albumin-to-alkaline phosphatase ratio; FBG, fasting blood glucose; CHD, coronary heart disease; DM, diabetes mellitus; ALT, alanine transaminase; AST, aspartate transaminase; RBP, retinol-binding protein; UA, uric acid; BUN, blood urea nitrogen; Scr, serum creatinine.

**Table 6:** Association of severe degeneration and non-fusion with AAPR.

Variable	Cases	Model 1 (Degeneration Model)		Model 2 (Non-Fusion Model)	
		OR [95% CI]	*p* for Trend	OR [95% CI]	*p* for Trend
AAPR (Median [Range])					
Q1 (0.42 [≤0.49])	82	Reference		Reference	
Q2 (0.56 [0.49–0.61])	81	0.632 [0.306–1.306]		0.653 [0.320–1.334]	
Q3 (0.67 [0.61–0.75])	82	0.731 [0.342–1.563]		0.400 [0.184–0.873]	
Q4 (0.85 [>0.75])	81	0.316 [0.139–0.719]	0.010	0.103 [0.038–0.277]	<0.001

AAPR, albumin-to-alkaline phosphatase ratio.

**Table 7:** Univariate and multivariate analysis model 2 of risk factors for non-fusion.

Variables	Univariate		Multivariate	
	OR (95% CI)	*p*	OR (95% CI)	*p*
Age (year)	0.995 (0.977–1.013)	0.569	0.987 (0.960–1.014)	0.345
Gender (male)	0.630 (0.385–1.031)	0.066	0.570 (0.261–1.247)	0.159
BMI	0.973 (0.909–1.042)	0.432	0.959 (0.880–1.046)	0.345
Smoking	1.086 (0.538–2.191)	0.819	1.915 (0.743–4.936)	0.179
Alcohol abuse	0.826 (0.337–2.023)	0.675	0.808 (0.262–2.489)	0.710
Hypertension	0.855 (0.530–1.379)	0.520	1.101 (0.582–2.081)	0.767
DM	0.742 (0.385–1.432)	0.374	0.666 (0.237–1.873)	0.441
CHD	0.470 (0.200–1.102)	0.082	0.357 (0.128–0.998)	0.049
Osteoporosis	1.222 (0.746–2.002)	0.426	1.129 (0.591–2.156)	0.713
Calcification	1.581 (0.977–2.559)	0.062	0.834 (0.439–1.586)	0.580
AAPR	0.009 (0.002–0.047)	<0.001	0.003 (0.0003–0.022)	<0.001
Calcium	1.616 (0.144–18.189)	0.697	0.397 (0.017–9.500)	0.569
phosphorus	9.892 (2.270–43.106)	0.002	16.677 (2.794–99.552)	0.002
FBG	1.024 (0.873–1.201)	0.770	1.104 (0.868–1.405)	0.418
BUN	0.985 (0.859–1.128)	0.823	1.007 (0.837–1.212)	0.938
Scr	0.988 (0.976–1.001)	0.062	0.996 (0.976–1.016)	0.673
UA	1.001 (0.998–1.004)	0.531	1.005 (1.001–1.010)	0.014
ALT	1.002 (0.986–1.018)	0.808	0.988 (0.952–1.025)	0.510
AST	1.014 (0.987–1.041)	0.318	1.016 (0.959–1.076)	0.595
RBP	0.988 (0.963–1.014)	0.356	0.986 (0.956–1.016)	0.344

BMI, body mass index; AAPR, albumin-to-alkaline phosphatase ratio; FBG, fasting blood glucose; CHD, coronary heart disease; DM, diabetes mellitus; ALT, alanine transaminase; AST, aspartate transaminase; RBP, retinol-binding protein; UA, uric acid; BUN, blood urea nitrogen; Scr, serum creatinine.

## Data Availability

The datasets generated and/or analyzed during the present study are available from the corresponding author upon reasonable request.

## Supplementary Materials

The following supporting information can be downloaded at: https://www.mdpi.com/article/10.3390/jcm11164719/s1, Table S1. Univariate and multivariate analysis model 3 (ALP) of risk factors for severe degeneration. Table S2. Univariate and multivariate analysis model 4 (ALB) of risk factors for severe degeneration. Table S3. Univariate and multivariate analysis model 5 (ALP) of risk factors for non-fusion. Table S4. Univariate and multivariate analysis model 6 (ALB) of risk factors for non-fusion.
