# Supplementary material for: Albumin-to-Alkaline Phosphatase Ratio as a Prognostic Biomarker for Spinal Fusion in Lumbar Degenerative Diseases Patients Undergoing Lumbar Spinal Fusion
Source: J Clin Med. 2022 Aug 12;11(16):4719. doi: 10.3390/jcm11164719 (PMC9409976; doi:10.3390/jcm11164719)
Supplement: Supplementary file 1 [file jcm-11-04719-s001.zip › jcm-1828802-supplementary.pdf]

Table S1. Univariate and multivariate analysis model 3 (ALP) of risk factors for severe degeneration.

Variables	Univariate		Multivariate	
	OR (95% CI)	p	OR (95% CI)	p
Age(year)	1.042(1.023-1.062)	< 0.001	1.027 (0.999-1.055)	0.056
Gender(male)	0.878(0.565-1.364)	0.562	0.998(0.504-1.978)	0.996
BMI	0.971(0.913-1.034)	0.358	0.974(0.900-1.055)	0.524
Smoking	1.124(0.587-2.151)	0.724	1.375(0.579-3.262)	0.470
Alcohol abuse	1.865(0.837-4.155)	0.127	1.992(0.732-5.419)	0.177
Hypertension	1.574(1.015-2.443)	0.043	1.139(0.632-2.055)	0.664
DM	1.117(0.630-1.982)	0.704	0.898(0.385-2.095)	0.804
CHD	0.789(0.402-1.548)	0.490	0.376(0.163-0.869)	0.022
Osteoporosis	1.734(1.096-2.742)	0.019	1.070(0.599-1.912)	0.820
Calcification	4.797(2.993-7.689)	< 0.001	3.445(1.950-6.085)	< 0.001
ALP	1.020(1.010-1.030)	< 0.001	1.011(0.999-1.022)	0.079
Calcium	6.241(0.649-59.996)	0.113	10.549(0.610-182.457)	0.105
phosphorus	0.618(0.166-2.308)	0.474	1.405(0.286-6.895)	0.675
FBG	1.096(0.944-1.274)	0.229	1.080(0.868-1.342)	0.491
BUN	0.988(0.873-1.118)	0.850	0.971(0.820-1.151)	0.737
Scr	1.001(0.992-1.010)	0.839	1.002(0.987-1.018)	0.757
UA	0.999(0.996-1.002)	0.472	1.001(0.997-1.004)	0.802
ALT	0.993(0.977-1.009)	0.405	0.999(0.964-1.035)	0.934
AST	1.011(0.985-1.038)	0.408	0.998(0.943-1.056)	0.950
RBP	0.960(0.937-0.984)	0.001	0.949(0.920-0.978)	0.001

BMI, body mass index; AAPR, albumin-to-alkaline phosphatase ratio; FBG, fasting blood glucose; CHD, coronary heart disease; DM, diabetes mellitus; ALT, alanine transaminase; AST, aspartate transaminase; RBP, retinol-binding protein; UA, uric acid; BUN, blood urea nitrogen; Scr, serum creatinine.

Table S2. Univariate and multivariate analysis model 4 (ALB) of risk factors for severe degeneration.

Variables	Univariate		Multivariate	
	OR (95% CI)	p	OR (95% CI)	p
Age(year)	1.042(1.023-1.062)	< 0.001	1.023(0.996-1.051)	0.096
Gender(male)	0.878(0.565-1.364)	0.562	1.006(0.508-1.993)	0.987
BMI	0.971(0.913-1.034)	0.358	0.974(0.899-1.054)	0.512
Smoking	1.124(0.587-2.151)	0.724	1.360(0.578-3.199)	0.481
Alcohol abuse	1.865(0.837-4.155)	0.127	2.085(0.774-5.618)	0.146
Hypertension	1.574(1.015-2.443)	0.043	1.133(0.631-2.036)	0.675
DM	1.117(0.630-1.982)	0.704	0.858(0.369-1.993)	0.722
CHD	0.789(0.402-1.548)	0.490	0.432(0.192-0.973)	0.043
Osteoporosis	1.734(1.096-2.742)	0.019	1.099(0.617-1.958)	0.748
Calcification	4.797(2.993-7.689)	< 0.001	4.300(2.542-7.273)	< 0.001
ALB	0.986(0.922-1.053)	0.671	0.976(0.889-1.072)	0.612
Calcium	6.241(0.649-59.996)	0.113	15.194(0.642-359.720)	0.092
phosphorus	0.618(0.166-2.308)	0.474	1.164(0.238-5.693)	0.852
FBG	1.096(0.944-1.274)	0.229	1.098(0.886-1.362)	0.392
BUN	0.988(0.873-1.118)	0.850	0.979(0.825-1.161)	0.806
Scr	1.001(0.992-1.010)	0.839	0.999(0.984-1.015)	0.910
UA	0.999(0.996-1.002)	0.472	1.001(0.997-1.004)	0.785
ALT	0.993(0.977-1.009)	0.405	0.995(0.961-1.031)	0.799
AST	1.011(0.985-1.038)	0.408	1.007(0.951-1.066)	0.812
RBP	0.960(0.937-0.984)	0.001	0.952(0.923-0.982)	0.002

BMI, body mass index; AAPR, albumin-to-alkaline phosphatase ratio; FBG, fasting blood glucose; CHD, coronary heart disease; DM, diabetes mellitus; ALT, alanine transaminase; AST, aspartate transaminase; RBP, retinol-binding protein; UA, uric acid; BUN, blood urea nitrogen; Scr, serum creatinine.

Table S3. Univariate and multivariate analysis model 5 (ALP) of risk factors for non-fusion

Variables	Univariate		Multivariate	
	OR (95% CI)	p	OR (95% CI)	p
Age(year)	0.995(0.977-1.013)	0.569	0.995(0.968-1.023)	0.736
Gender(male)	0.630(0.385-1.031)	0.066	0.607(0.276-1.336)	0.215
BMI	0.973(0.909-1.042)	0.432	0.959(0.878-1.046)	0.345
Smoking	1.086(0.538-2.191)	0.819	1.944(0.751-5.034)	0.171
Alcohol abuse	0.826(0.337-2.023)	0.675	0.672(0.206-2.195)	0.511
Hypertension	0.855(0.530-1.379)	0.520	1.076(0.565-2.048)	0.824
DM	0.742(0.385-1.432)	0.374	0.635(0.219-1.840)	0.403
CHD	0.470(0.200-1.102)	0.082	0.284(0.093-0.868)	0.027
Osteoporosis	1.222(0.746-2.002)	0.426	1.043(0.544-1.998)	0.899
Calcification	1.581(0.977-2.559)	0.062	0.741(0.386-1.423)	0.368
ALP	1.034(1.022-1.046)	< 0.001	1.047(1.031-1.063)	< 0.001
Calcium	1.616(0.144-18.189)	0.697	0.230(0.009-5.732)	0.370
phosphorus	9.892(2.270-43.106)	0.002	22.097(3.606-135.393)	0.001
FBG	1.024(0.873-1.201)	0.770	1.094(0.852-1.405)	0.480
BUN	0.985(0.859-1.128)	0.823	1.028(0.854-1.236)	0.773
Scr	0.988(0.976-1.001)	0.062	0.997(0.976-1.017)	0.740
UA	1.001(0.998-1.004)	0.531	1.005(1.001-1.010)	0.020
ALT	1.002(0.986-1.018)	0.808	0.996(0.960-1.034)	0.842
AST	1.014(0.987-1.041)	0.318	0.999(0.943-1.058)	0.960
RBP	0.988(0.963-1.014)	0.356	0.979(0.950-1.009)	0.170

BMI, body mass index; AAPR, albumin-to-alkaline phosphatase ratio; FBG, fasting blood glucose; CHD, coronary heart disease; DM, diabetes mellitus; ALT, alanine transaminase; AST, aspartate transaminase; RBP, retinol-binding protein; UA, uric acid; BUN, blood urea nitrogen; Scr, serum creatinine.

Table S4. Univariate and multivariate analysis model 6 (ALB) of risk factors for non-fusion

Variables	Univariate		Multivariate	
	OR (95% CI)	p	OR (95% CI)	p
Age(year)	0.995(0.977-1.013)	0.569	0.988(0.962-1.014)	0.351
Gender(male)	0.630(0.385-1.031)	0.066	0.723(0.352-1.485)	0.377
BMI	0.973(0.909-1.042)	0.432	0.953(0.880-1.032)	0.239
Smoking	1.086(0.538-2.191)	0.819	1.614(0.677-3.849)	0.280
Alcohol abuse	0.826(0.337-2.023)	0.675	1.035(0.374-2.865)	0.948
Hypertension	0.855(0.530-1.379)	0.520	1.046(0.575-1.906)	0.882
DM	0.742(0.385-1.432)	0.374	0.673(0.267-1.698)	0.401
CHD	0.470(0.200-1.102)	0.082	0.486(0.188-1.256)	0.137
Osteoporosis	1.222(0.746-2.002)	0.426	1.299(0.715-2.358)	0.390
Calcification	1.581(0.977-2.559)	0.062	1.853(1.068-3.213)	0.028
ALB	1.014(0.943-1.091)	0.701	1.020(0.927-1.122)	0.685
Calcium	1.616(0.144-18.189)	0.697	0.239(0.009-6.250)	0.390
phosphorus	9.892(2.270-43.106)	0.002	9.005(1.761-46.050)	0.008
FBG	1.024(0.873-1.201)	0.770	1.144(0.910-1.437)	0.249
BUN	0.985(0.859-1.128)	0.823	1.054(0.886-1.254)	0.552
Scr	0.988(0.976-1.001)	0.062	0.986(0.967-1.004)	0.129
UA	1.001(0.998-1.004)	0.531	1.004(1.0002-1.008)	0.038
ALT	1.002(0.986-1.018)	0.808	0.984(0.950-1.019)	0.372
AST	1.014(0.987-1.041)	0.318	1.028(0.973-1.086)	0.329
RBP	0.988(0.963-1.014)	0.356	0.985(0.955-1.015)	0.324

BMI, body mass index; AAPR, albumin-to-alkaline phosphatase ratio; FBG, fasting blood glucose; CHD, coronary heart disease; DM, diabetes mellitus; ALT, alanine transaminase; AST, aspartate transaminase; RBP, retinol-binding protein; UA, uric acid; BUN, blood urea nitrogen; Scr, serum creatinine.
